# Mass closure vs. layer-by-layer closure of transverse laparotomy in children: a randomized trial with ultrasound and elastography outcomes

**DOI:** 10.3389/fped.2025.1655851

**Published:** 2025-08-13

**Authors:** Mohamed Ali Shehata, Mohammad Gharieb Khirallah, Mazin Kurdi, Shaimaa Abdelmonem Abdelwahab, Radwa Elkhouly, Suzan Ezzat Gado

**Affiliations:** ^1^ Pediatric Surgery Unit, Department of Surgery, Tanta University, Tanta, Egypt; ^2^ Pediatric Surgery Unit, Department of Surgery, King Abdulaziz University Hospital, Jeddah, KSA; ^3^ Rheumatology, Physical Medicine and Rehabilitation Department, Tanta University, Tanta, Egypt

**Keywords:** transverse laparotomy, children, mass closure, layer-by-layer closure, elastography

## Abstract

**Background:**

transverse laparotomy incision presents a common and widely used one in infants and children. The fascial incision is either closed in one mass or layer-by-layer closure. Both methods nearly have the same outcomes. This study randomly compared the two main techniques of closure of the transverse laparotomy incision, regarding clinical, sonographic, and elastrographic changes.

**Methods:**

This trial included infants and children who were subjected to a transverse upper abdominal laparotomy incision. Patients whose muscle sheath complex defects were closed using the mass closure technique were allotted to **Group A**. Patients whose defects were closed using the layer-by-layer closure technique were allotted to **Group B.** The wounds were subjected to musculoskeletal ultrasound and elastography examination.

**Results:**

The age was 22.3 months and 22.5 months in Group **A** and Group **B**, respectively. Fatty infiltration >30% but <40% was observed in 35 cases in Group **A** and 15 cases in Group **B**, (*p* = 0.04). The 20%–40% fibrosis in the muscle sheath complex observed in 33 cases in Group **A** and 9 cases in Group **B**, (*p* = 0.02). During the last follow-up examination at the end of first year, the shear wave speed was 6.4 m/s in Group **A** and 3.1 m/s in Group **B** (*p* = 0.05).

**Conclusion:**

Mass closure resulted in significantly higher stiffness and fatty infiltration of the muscle sheath complex compared to layer-by-layer closure, as shown by elastography and ultrasound. These biomechanical alterations may predispose to increased long-term risk of incisional hernia despite comparable short-term outcomes.

## Introduction

Transverse laparotomy is commonly employed in pediatric surgery due to its suitability for the barrel-shaped abdomen of infants and young children and its perceived lower risk of incisional hernia compared to midline incisions. However, the optimal technique for closing such incisions remains a subject of ongoing clinical debate, with limited consensus regarding long-term outcomes (1, 2). However, the choice between mass closure and layer-by-layer closure techniques continues to divide surgeons, with conflicting evidence regarding their long-term outcomes (3–5).

The clinical dilemma stems from the lack of high-quality, randomized trials comparing these techniques, particularly in pediatric populations. While some studies suggest that mass closure reduces operative time and may lower the risk of wound complications (6, 7), others advocate for layer-by-layer closure, citing better anatomical restoration and potentially lower incisional hernia rates (8, 9). A meta-analysis by Henriksen et al. highlighted the inconsistency in existing literature, noting the absence of long-term data on functional outcomes and tissue healing properties (10). Furthermore, most studies focus solely on short-term complications, such as wound dehiscence or infection, neglecting critical long-term metrics like tissue stiffness, fatty infiltration, and the biomechanical integrity of the abdominal wall (11, 12).

This gap in knowledge is particularly concerning given the lifelong implications of incisional hernias and abdominal wall dysfunction in children. Recent advances in imaging technologies, such as musculoskeletal ultrasound (MSU) and shear wave elastography (SWE), offer new opportunities to objectively assess tissue healing and biomechanical properties post-repair (13, 14). These tools can provide insights into the morphological and functional changes that precede clinical complications, enabling early intervention and improved prognostic accuracy.

### Study objectives

This randomized trial aims to compare mass closure and layer-by-layer closure of transverse laparotomy incisions in children, with quantitative assessment of tissue stiffness using SWE, degree of fatty infiltration and fibrosis via MSU, and operative time differences between the two techniques.

By integrating clinical, sonographic, and elastographic outcomes, this study seeks to address the existing literature gaps and provide evidence-based recommendations for pediatric abdominal wall closure.

## Methods

### Study design

This was a **prospective, randomized, single-center, two-arm parallel-group trial** comparing mass closure vs. layer-by-layer closure of transverse laparotomy incisions in children between January 2022 and January 2024. The study was registered at **ClinicalTrials.gov** (NCT06016426) and approved by the **Institutional Ethics Committee** of faculty of Medicine, Tanta university, Tanta, Egypt with number (36264PR286/8/23). Written informed consent was obtained from parents or legal guardians.

### Participants

#### Inclusion criteria

• Children aged ≤5 years.
• No prior abdominal surgeries.
• Elective or emergency transverse laparotomy (upper abdominal). The most common surgical indications included elective procedures such as choledochal cyst excision, duodenal atresia repair, Wilms tumor, medulloblastoma, pyloric stenosis, and hepaticojejunostomy, as well as emergency conditions including volvulus due to malrotation, intestinal atresia, and small bowel obstruction.

#### Exclusion criteria

• Prematurity (<37 weeks gestational age).
• Congenital abdominal wall defects (e.g., gastroschisis, omphalocele).
• Severe comorbidities (cardiac, hepatic, respiratory, or nutritional disorders).
• Peritonitis or generalized intra-abdominal infection at the time of surgery.

### Randomization and allocation concealment

Patients were randomized into two groups using a computer-generated block randomization sequence with variable block sizes to ensure balanced group allocation. Allocation concealment was maintained using sequentially numbered, opaque, sealed envelopes (SNOSE) opened only in the operating room by a nurse not involved in patient care or outcome assessment.

• **Group A**: Mass closure of the muscle sheath complex (MSC)
• **Group B**: Layer-by-layer closure of the MSC

**Figure 1:**
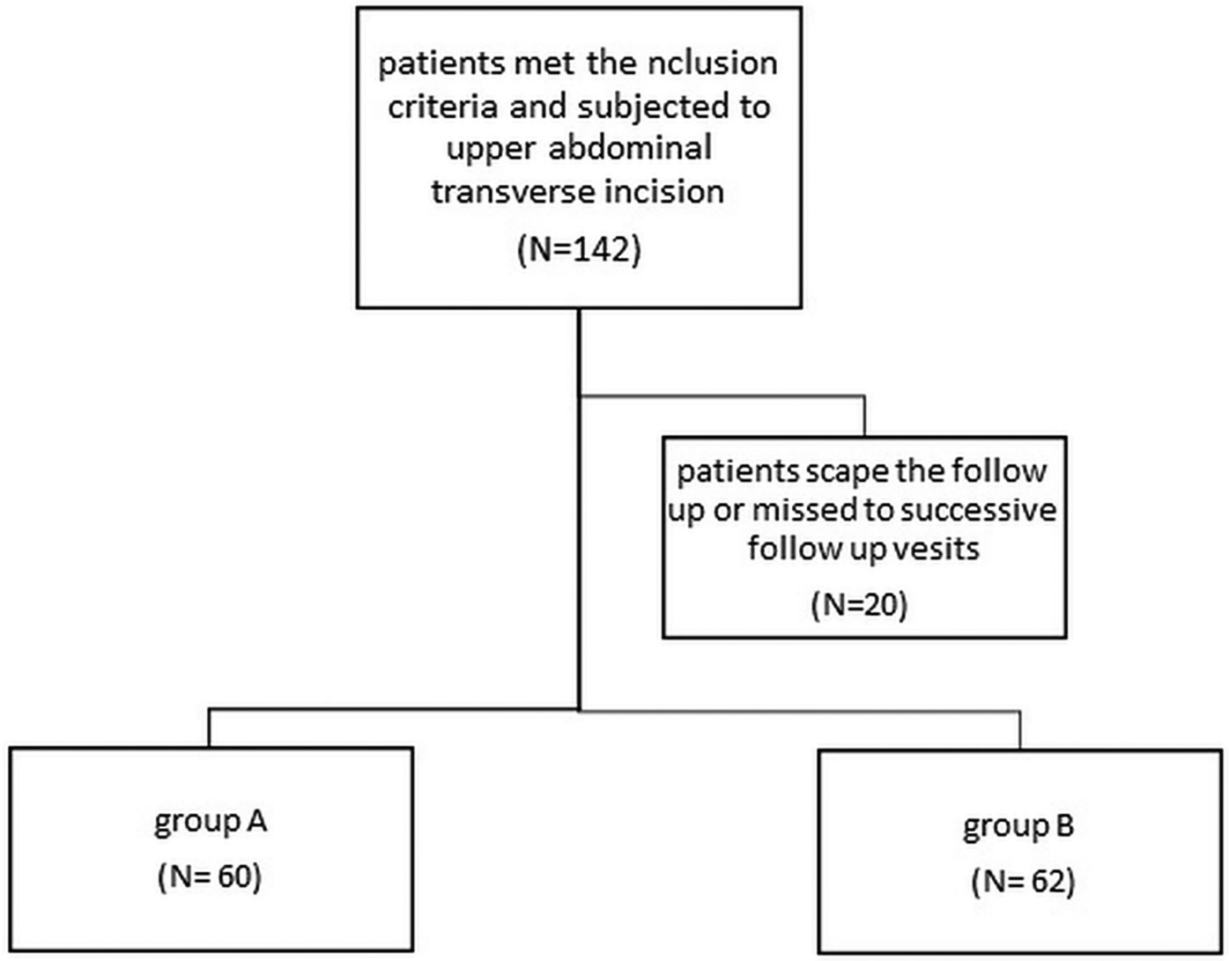
Flowchart of the patients in the study.

A total of 157 eligible patients were randomized; 142 met the inclusion criteria, and 122 completed the full follow-up period (Figure 1).

### Blinding

While blinding of the operating surgeon was not feasible due to the nature of the intervention, all postoperative outcome assessors, including radiologists performing musculoskeletal ultrasound (MSU) and shear wave elastography (SWE), were blinded to both group allocation and clinical outcomes, including surgical technique, patient history, and physical examination findings, to minimize assessment bias.

### Surgical techniques

All operations were performed by senior pediatric surgeons following a standardized protocol. After the principal procedure, the wound was irrigated with warm saline.

• **Mass Closure (Group A)**: A continuous single-layer closure using Vicryl 2/0® with a round needle, incorporating the peritoneum, fascia transversalis, posterior and anterior rectus sheaths, and external oblique aponeurosis. Small bites were taken 5 mm from the fascial edge and spaced 5 mm apart to ensure adequate perfusion and minimize tissue ischemia.
• **Layer-by-Layer Closure (Group B)**: The peritoneum, fascia transversalis, and posterior rectus sheath were closed together as one layer using continuous Vicryl 2/0® sutures. The anterior rectus sheath was closed separately with a second continuous suture. Both layers adhered to the same small-bite technique.

### Postoperative follow-up and imaging

The patients in both groups were scheduled for postoperative follow-up. The wounds were examined weekly for 1 month. The primary aim of this examination was to detect the occurrence of wound dehiscence, wound infection, or seroma. Following this, the wound was examined monthly for incisional hernia development for the next 1 year.

Patients who developed complete wound dehiscence or incisional hernia during the first 3 months postoperatively or later were excluded from the follow-up process.

Furthermore, the wound was subjected to musculoskeletal ultrasound (MSU) and elastography examination. MSU and SWE examinations were performed at 3, 6, 9, and 12 months postoperatively, aligning with clinical follow-up visits to assess progressive tissue changes over time. All patients underwent sonographic examinations using UGEO H60® (Samsung Medison) with linear array transducers (frequencies range, 9–13 MHz) at the Ultrasound Unit of the Rheumatology and Rehabilitation Department.

The MSU and elastography assessment spotlight on the MSC consisted of the rectus abdominis muscle, three lateral muscles (the external oblique, internal oblique, and transversus abdominis muscles), and the deep layer of extraperitoneal fat.

While the patient was in the supine position, the ultrasound transducer was placed in the transverse orientation and perpendicular to the skin at the site of the transverse scar with the least amount of pressure possible. The examination was repeated on the contralateral side of the anterior abdominal wall at the scar level to compare the affected side with the unaffected side.

Shear wave elastography measurements of the abdominal wall laparotomy incision were obtained using Mindray Ultrasound equipped with a linear array transducer ranging from 4 to 9 MHz in frequency. The probe was applied perpendicular to the skin and parallel to the muscle fibres. The elastography box was adjusted to the appropriate size, wherein the width was adjusted to the maximum depth of the deep surface of the abdominal wall.

**Figure 2:**
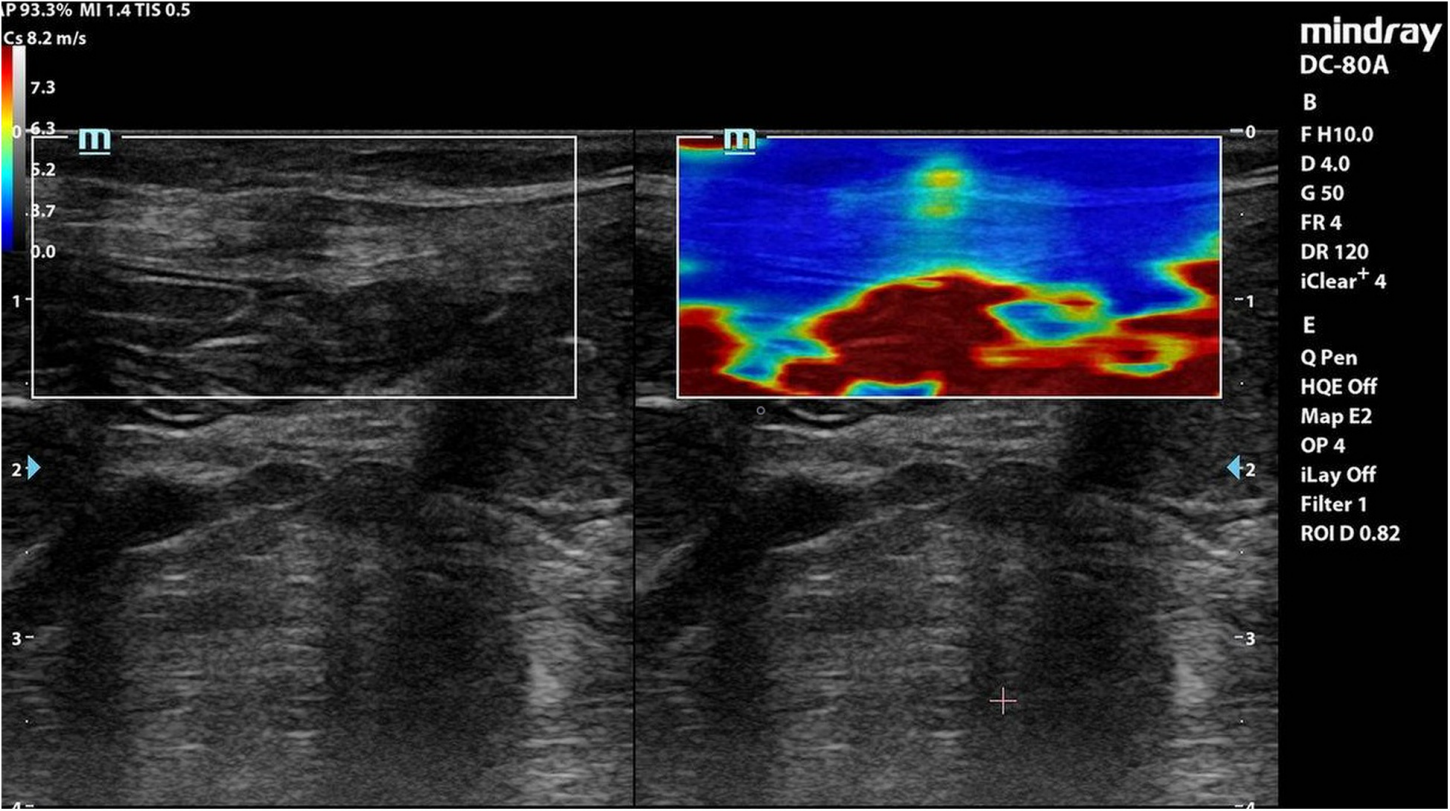
Elastography of muscle sheath complex of a patient in **group A** showed increased ratio of red colour (increased stiffness).

**Figure 3:**
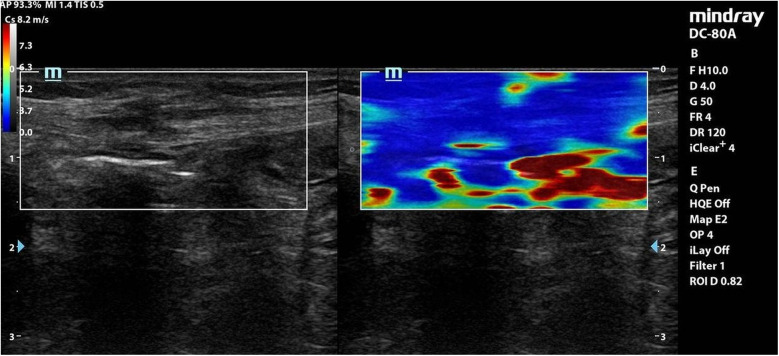
Elastography of muscle sheath complex of a patient in **group B** showed increased ratio of blue colour (decreased stiffness).

**Figure 4:**
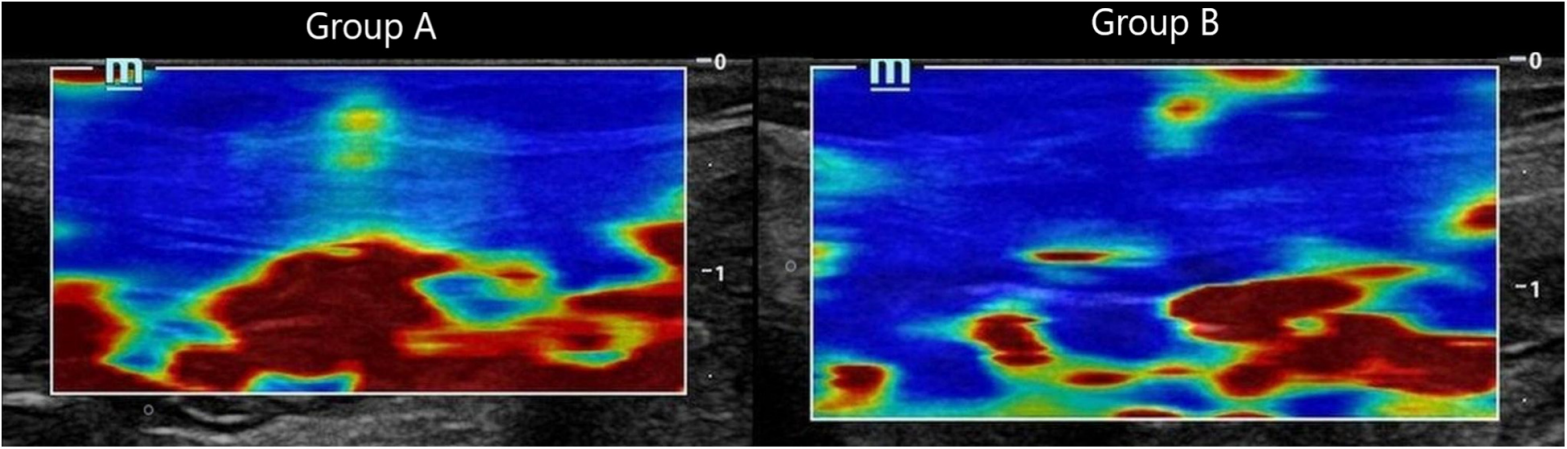
Composite panel of shear wave elastography images showing the muscle sheath complex in Group A (mass closure, left) and Group B (layered closure, right). Red coloration in Group A reflects increased stiffness, while the blue-dominated image in Group B indicates preserved tissue elasticity.

Each coloured pixel represented the estimated shear wave speed (SWS) at the site where the probe was applied. Red pixels represented an increased stiffness of the tissues, while the blue pixels represented elastic regions (Figures 2–4).

A sampling of these boxes was then placed onto the images to obtain a quantitative measurement of the SWS reported in metres per second. Higher SWS correlated with increased tissue stiffness, whereas slower SWS correlated with elastic soft tissues.

**Figure 5:**
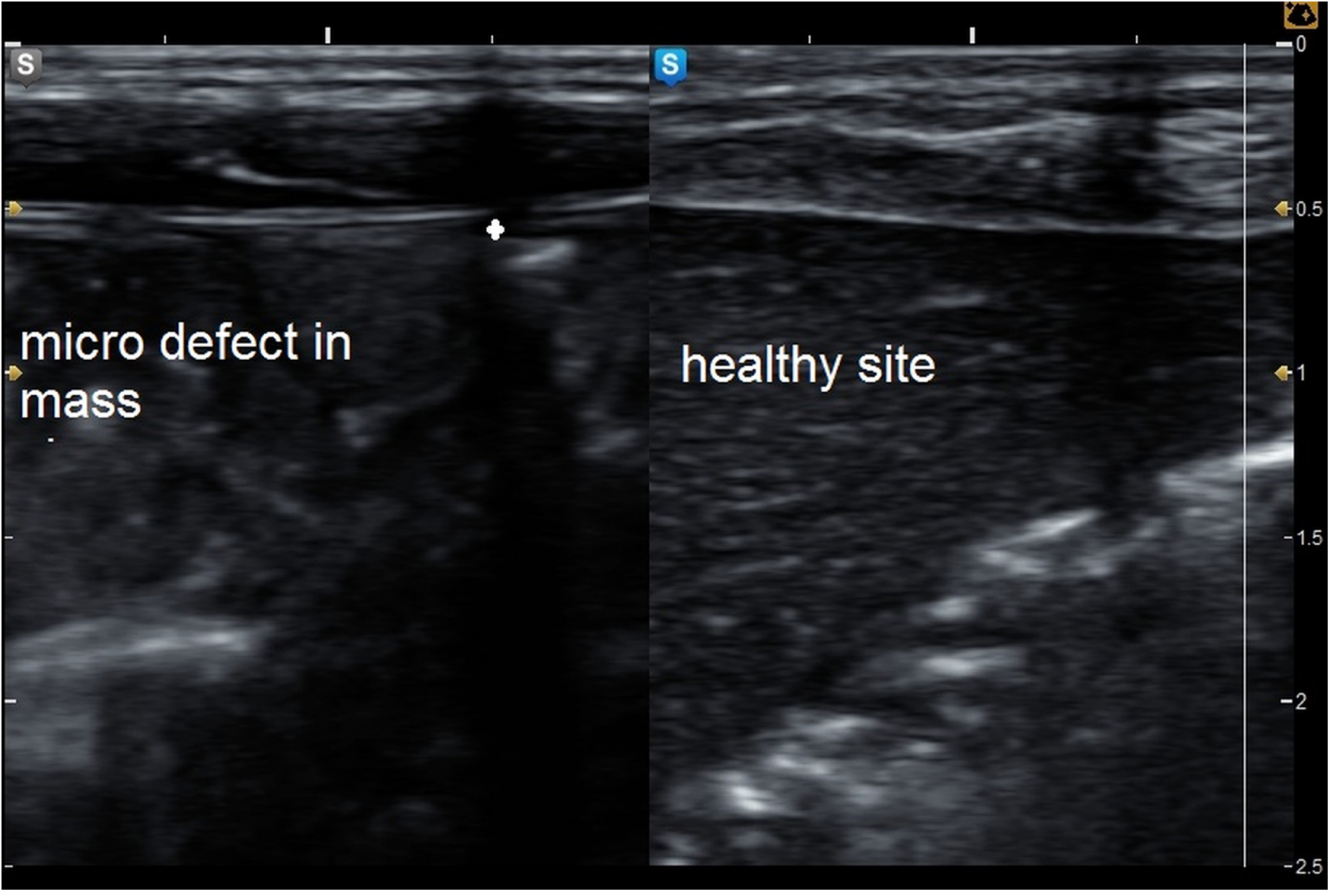
Grey mode ultrasound showed micro defect in the muscle sheath complex with comparison to the healthy side of the abdominal wall.

**Figure 6:**
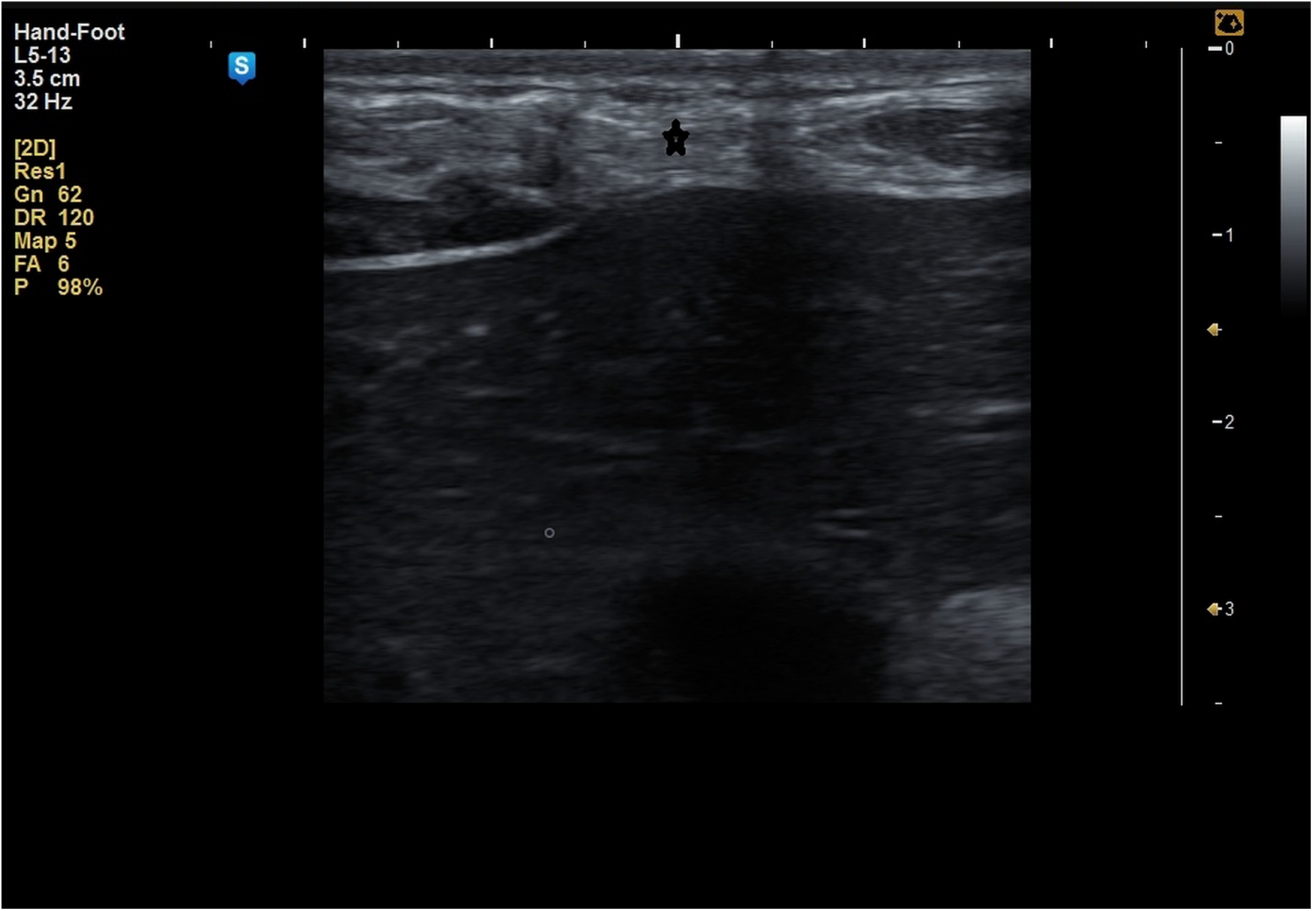
Fatty infiltration of the muscle sheath complex (hyper echoic lesions) marked by * in a patient of **group A**.

**Figure 7:**
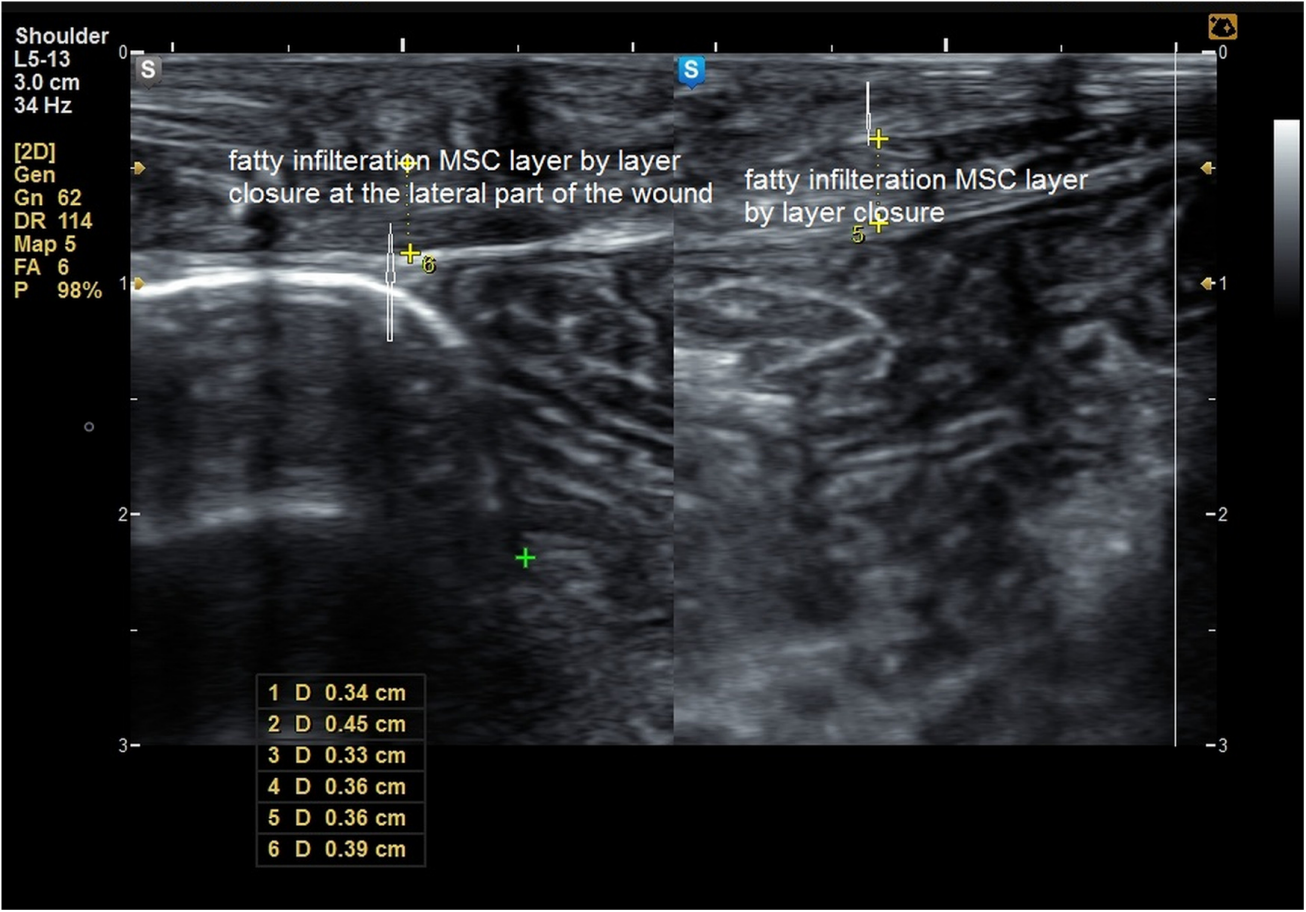
Fatty infiltration of the muscle sheath complex in a patient in **group B**.

**Figure 8:**
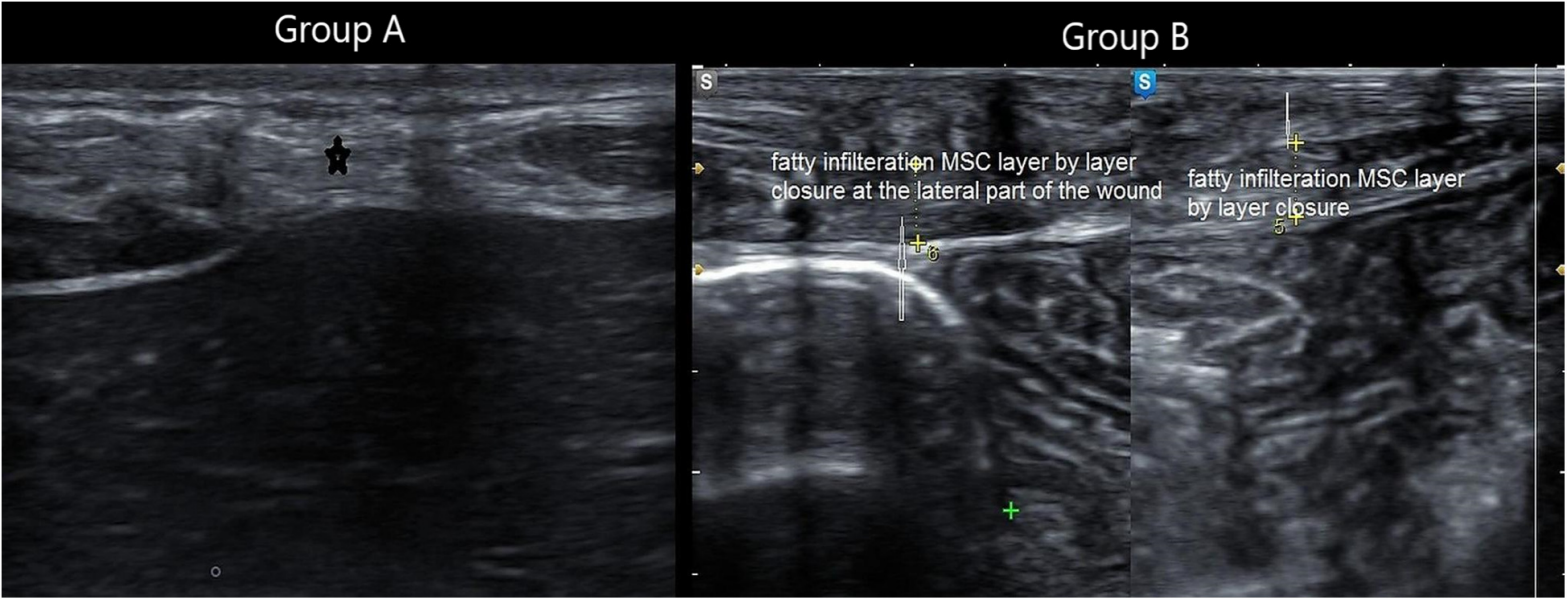
Composite panel of musculoskeletal ultrasound showing fatty infiltration (white hyperechoic areas) in the muscle sheath complex of a patient in Group A, compared to minimal infiltration in Group B.

The MSU examination delineated the thickness of the MSC compared to the healthy side, degree of fibrosis, presence of any undetected clinical defects, and degree of fatty infiltration of the **MSC** (Figures 5–8).

Inter-observer and intra-rater variability were not formally assessed. However, all imaging was performed by experienced radiologists following a standardized protocol to reduce the potential for measurement bias.

### Outcome measures

#### Primary endpoint

• Incisional hernia rates at 12 months.

#### Secondary endpoints

• Tissue stiffness (SWE).
• Fatty infiltration/fibrosis (MSU).
• Operative time.

### Sample size and power analysis

Sample size was calculated using G*Power 3.1.9.7. To detect a medium effect size (Cohen's *d* = 0.5) with 80% power at a 5% significance level, a minimum of 104 patients (52 per group) was required. Allowing for a 15% dropout rate, a total of 122 patients were followed up and included in the final analysis.

### Statistical analysis

Statistical analyses were performed using IBM SPSS version 21. Continuous variables were expressed as mean ± standard deviation and compared using the independent *t*-test. Categorical variables were analyzed using the chi-square test. A *p*-value <0.05 was considered statistically significant. Confidence intervals (95%) were calculated for key outcomes and are reported alongside *p*-values in the Results section.

**N.B.** This study adheres to the CONSORT guidelines for randomized controlled trials. A completed CONSORT checklist is provided in the Supplementary Appendix (Supplementary Table S1).

## Results

### Patient demographics and baseline characteristics

Among 387 infants and children who underwent laparotomy, 142 fulfilled the inclusion criteria for this study. These patients were randomly categorised into **Group A:** mass closure, (*n* = 79); **Group B:** layer-by-layer closure, (*n* = 78), with **122 patients** completing the 12-month follow-up, 60 in **Group A** and 62 in **group B** (Figure 1). The groups were well-matched at baseline (Table 1).

**Table 1:** Demographic and preoperative data.

Parameters	Group A (*N* = 60)	Group B (*N* = 62)	*p*-value	95% CI (A vs. B)
Age (months ± SD)	22.3 ± 11.3	23.5 ± 12.2	0.254	[−3.3, 1.0]
Sex				
Males	32 (53.3%)	35 (56.5%)	0.371	[−12.3%, 10.2%]
Females	28 (46.7%)	27 (43.5%)	0.361	
Body weight (kg ± SD)	15.2 ± 7.1	17.4 ± 8.5	0.249	[−4.98, 0.58]
Albumin (gm/dl ± SD)	4.1 ± 0.5	3.9 ± 0.4	0.152	[0.04, 0.36]
Prealbumin (mg/dl ± SD)	19.5 ± 1.2	20.02 ± 0.9	0.534	[−0.90, −0.14]
Rt. Side incision (No. + %)	52 (86.7%)	51 (82.3%)	0.245	[−5.0%, 13.0%]
Lt. side incision (No. + %)	8 (13.3%)	11 (17.7%)	0.234	[−3.1%, 10.3%]
Elective surgery (No. + %)	50 (83.3%)	53 (85.5%)	0.587	[−6.2%, 11.2%]
Emergency surgery (No. + %)	10 (16.7%)	9 (14.5%)	0.424	[−5.7%, 9.9%]

CI, Confidence interval.

The mean age was 22.3 months and 23.5 months in Group A and B, respectively. There were 32 males in Group A and 35 in Group B. The mean body weight was 15.2 kg and 17.4 kg in Group A and B, respectively. The preoperative serum albumin and prealbumin levels were 4.1 g/dl and 19.5 mg/dl in Group A and 3.9 g/dl and 20.02 mg/dl in Group B, with no statistically significant differences in either parameter between the groups. Elective surgery was performed for 50 patients (83.3%) in Group A and 53 patients (85.5%) in Group B. Emergency procedures accounted for 16.7% and 14.5% of cases, respectively, with no significant difference between groups (*p* = 0.424). This comparable distribution suggests that the differences in postoperative wound characteristics, such as stiffness and fatty infiltration, are unlikely to be confounded by surgical urgency or baseline severity of patient condition. Importantly, patients with peritonitis, extreme prematurity, or severe comorbidities were excluded from the study, minimizing confounding due to underlying illness severity. As most pediatric abdominal surgeries are performed electively, the cohort primarily reflects standard-risk cases, with well-balanced surgical urgency between groups.

### Operative outcomes

The mean length of the transverse laparotomy incision was 12.2 cm in Group A and 13.4 cm in Group B, with no statistically significant difference. In Group A, 42 patients (70%) had incisions between 10 and 13 cm, and 15 patients (25%) had incisions >13 cm. In Group B, 40 patients (65%) had incisions between 10 and 13 cm, and 18 patients (29%) had incisions >13 cm. Only a few patients in each group had incisions <10 cm or >16 cm, typically in smaller infants or more extensive procedures, respectively (Table 2).

**Table 2:** Operative and postoperative data.

Parameter		Group A (*N* = 60)	Group B (*N* = 62)	*p* value	95% CI
Mean length of wound (cm ± SD)		12.2 ± 1.5	13.4 ± 1.8	0.731	[−1.79, −0.61]
Distribution of wound length	<10 cm	3 (5.0%)	2 (3.2%)	0.621	[−5.3%, 8.8%]
	10–13 cm	42 (70.0%)	40 (64.5%)	0.519	[−11.1%, 22.1%]
	>13–16 cm	15 (25.0%)	18 (29.0%)	0.616	[−19.8%, 11.7%]
	>16 cm	0 (0%)	2 (3.2%)	0.161	[−7.6%, 1.2%]
Mean operative time of MSC closure (min ± SD)		13.4 ± 2.4	20.5 ± 2.2	<0.0001*	[−7.92, −6.28]
Post-operative seroma (No. + %)		2 (3.3%)	8 (12.9%)	0.0453*	[−19.1%, −0.1%]
Postoperative wound infection (No. + %)		5 (8.3%)	5 (8.06%)	1.0	[−9.5%, 10.0%]
Wound dehiscence (No. + %)		2 (3.3%)	3 (4.8%)	1.0	[−8.5%, 5.5%]
Incisional hernia (No. + %)		7 (11.7%)	8 (12.9%)	1.0	[−12.9%, 10.4%]

* Significant.

CI, confidence interval.

The mean operative time was 13.4 min in Group A and 20.5 min in Group B, with a statistically significant difference (*p* < 0.0001). This difference could be attributed to the meticulous closure of each layer of the peritoneum and MSC in Group B. The single-layer mass closure of the peritoneum and MSC in Group A markedly reduced the operative time required to close the defect. The incidence of postoperative seroma was significantly higher in Group B than in Group A (12.9% vs. 3.3%; *p* = 0.0453). This could be because of dead space development between each closed layer of the MSC and the subsequent layers. Moreover, the layer-by-layer closure may theoretically interrupt the lymphatic vessels crossing the layers. No statistically significant differences in wound infection, wound dehiscence, or development of early postoperative incisional hernia were observed between the groups. At the 12-month follow-up, the incidence of incisional hernia was 11.7% in Group A and 12.9% in Group B. The difference was not statistically significant (*p* = 1.0).

### Musculoskeletal ultrasound (MSU) findings

During the follow-up of patients in both groups using MSU, the mean thickness of the MSC at the laparotomy site was 1.2 cm in Group A and 1.8 cm in Group B. Although Group B patients showed an increase in the mean thickness of the MSC compared to Group A, the difference was not statistically significant. During MSU examination of the MSC, fatty infiltration of this complex appeared as hyper-echoic lesions. The volume of these hyper-echoic lesions in relation to the volume of MSC was <20% in 10 cases (16.7%) in Group A and 37 cases (59.7%) in Group B, showing a statistically significant difference (*p* = 0.03). When the fatty infiltration was 20%–30%, no statistically significant difference was observed between the groups. Fatty infiltration >30% but <40% was observed in 35 cases in Group A and 15 cases in Group B, with a statistically significant difference (*p* = 0.04) (Table 3).

**Table 4:** Musculoskeletal ultrasound during the follow up period.

Timepoint (mean ± SD)	Group A (*N* = 60)	Group B (*N* = 62)	*p* value	95% CI (A vs. B)
At the end of 3rd month	2. 5 ± 0.2	1.9 ± 0.3	0.194	[−0.3, 1.5]
At the end of the 6th month	4.6 ± 0.5	2.1 ± 0.8	0.03*	[1.7, 3.6]
At the end of the 9th month	5.7 ± 0.7	3.1 ± 0.4	0.03*	[1.9, 3.4]
At the end of the 12th month	6.4 ± 0.5	3.1 ± 0.4	0.02*	[2.9, 3.8]

* Significant.

CI, confidence interval.

The degree of fibrosis within the MSC was also assessed. Fibrosis between 20%–40% was significantly more common in Group A (33 cases, 55%) compared to Group B (9 cases, 14.5%) (*p* = 0.02). Conversely, mild fibrosis (<10%) was more prevalent in Group B (39 cases, 62.9%) vs. Group A (12 cases, 20%) (*p* = 0.03). No statistically significant difference was observed in fibrosis levels between 10%–20%.

No statistically significant difference was observed in the number of cases with MSC defects detected on MSU between the groups.

### Shear wave elastography (SWE) outcomes

At the end of 3 months postoperatively, the mean SWS was 2.5 m/s and 1.9 m/s in Groups A and B, respectively, without a statistically significant difference. During the follow-up visit at the end of 6 months postoperatively, a significant increase in the SWS was observed (4.6 m/s in Group A vs. 2.1 m/s in Group B; *p* = 0.03). This reflected the increased stiffness of the MSC in the Group A patients. However, by 12 months postoperatively, the MSC in Group B demonstrated significantly greater elasticity, as reflected by a lower mean shear wave speed of 3.1 ± 0.4 m/s compared to 6.4 ± 0.5 m/s in Group A (*p* = 0.02). At the end of 9 months postoperatively, the SWS continued to accelerate in Group A (5.7 m/s), while that in Group B was 3.1 m/s, with a statistically significant difference. During the last follow-up examination at the end of 1 year, the SWS was 6.4 m/s in Group A and 3.1 m/s in Group B, with a statistically significant difference (*p* = 0.02). A progressive increase in the SWS was noted over time. This difference was more significant at the end of the follow-up period in Group A than in Group B (Table 4).

**Table 3:** Shear wave speed (SWS) in both groups during the postoperative follow up period.

MSC findings	Group A (*N* = 60)	Group B (*N* = 62)	*p* value	95% CI
The mean thickness of MSC at the site of laparotomy in (cm ± SD)	1.2 ± 0.23	1.8 ± 0.32	0.241	[−0.41, 0.17]
The degree of fatty infiltration MSC at the laparotomy incision				
<20% of	10 (16.7%)	37 (59.7%)	0.03*	[28.4%, 56.1%]
20%–30%	15 (25%)	10 (16.1%)	0.534	[−9.1%, 25.1%]
30%–40%	35 (58.3%	15 (24.2%)	0.04*	[12.2%, 49.3%]
The degree of fibrosis in MSC at the site of laparotomy				
<10%	12 (20%)	39 (62.9%)	0.03*	[24.1%, 58.5%]
10%–20%	15 (25%)	14 (22.6%)	0.271	[−12.7%, 18.2%]
20%–40%	33 (55%)	9 (14.5%)	0.02*	[21.3%, 58.9%]
Ultrasound detected defects in the MSC without a clinical evident hernia (No. + %)	5 (8.3%)	6 (9.7%)	0.615	[−7.8%, 10.4%]

* Significant.

CI, confidence interval; MSC, muscle sheath complex.

**Interpretation**: Group A exhibited **stiffer scars** (red-dominated elastography, Figure 2), while Group B retained **greater elasticity** (blue-dominated, Figure 3).

### Clinical hernia rates

No significant difference in **incisional hernia** at 12 months (8.8% vs. 10.2%, *p* = 0.192). However, SWE/MSU suggested **higher biomechanical** risk in Group A.

## Discussion

The optimal technique for closing transverse laparotomy incisions in children remains a contentious issue in pediatric surgery. While transverse incisions are widely preferred in infants and young children due to their anatomical suitability for the barrel-shaped abdomen, the debate over whether mass closure or layer-by-layer closure yields superior long-term outcomes persists. This randomized trial provides critical insights into the biomechanical and morphological differences between these two techniques, leveraging advanced imaging modalities such as musculoskeletal ultrasound (MSU) and shear wave elastography (SWE) to assess tissue healing beyond traditional clinical endpoints.

The structural integrity of the musculoskeletal complex (MSC) is fundamentally influenced by biomechanical forces acting upon it. According to **Laplace's law**, the tension on the wall of a cylindrical structure increases with internal pressure and radius and decreases with wall thickness. This principle helps explain why certain alterations in tissue geometry or pressure conditions may predispose to mechanical failure, such as hernia formation. Understanding these biomechanical underpinnings is essential to interpreting imaging findings and their clinical implications in our patient population (9, 14). This phenomenon has been documented in adult populations, where stiffer scar tissue correlates with higher hernia occurrence rates (9). In pediatric patients, whose abdominal walls are still developing, such biomechanical alterations could have even more profound implications for long-term structural integrity.

### Operative efficiency vs. long-term tissue integrity

One of the most striking findings of this study was the significant reduction in operative time with mass closure (13.4 min vs. 20.5 min, *p* < 0.0001), reinforcing prior evidence that single-layer closure is technically faster and logistically advantageous, particularly in emergency settings (6, 7). The small-bite technique (5 mm from the fascial edge, spaced 5 mm apart) was employed in both groups to minimize tissue ischemia and optimize perfusion, a strategy supported by studies demonstrating reduced fascial dehiscence with continuous suturing (15, 16). However, despite its efficiency, mass closure was associated with concerning long-term tissue changes, including increased stiffness (SWS: 6.4 m/s vs. 3.1 m/s, *p* = 0.02) and greater fatty infiltration (>30% in 58.3% of Group A vs. 24.2% in Group B, *p* = 0.04). These findings suggest that while mass closure expedites surgery, it may compromise the biomechanical integrity of the abdominal wall over time.

### Fibrosis, fatty infiltration, and hernia risk

A key distinction between the two techniques was the degree of fibrosis observed in Group A (55% vs. 14.5% in Group B, *p* = 0.02). This discrepancy likely stems from the nature of mass closure, where sutures traverse multiple tissue planes (peritoneum, fascia transversalis, and rectus sheaths), creating a dense fibrotic scar along the suture line. In contrast, layer-by-layer closure confines sutures to individual anatomical planes, potentially preserving the natural elasticity of each layer. Fibrosis not only increases tissue stiffness but may also impair vascularization and remodeling, further weakening the repair site (12, 14).

Fatty infiltration, another marker of impaired healing, was significantly more prevalent in Group A. This pathological fat deposition within the muscle sheath complex (MSC) could disrupt muscle function and contribute to progressive abdominal wall laxity, a known risk factor for incisional hernia formation (9). Although the 12-month clinical hernia rates did not differ significantly (11.7% vs. 12.9%, *p* = 1.0), the higher incidence of subclinical micro-defects detected via MSU in both groups (11 cases total) suggests that structural deterioration may precede palpable hernia development. These subclinical MSC defects, while not immediately clinically apparent, may represent early markers of biomechanical wall failure. Progressive changes such as micro-defects, fibrotic remodeling, and fatty infiltration may serve as precursors to overt hernia formation. Detecting these changes early through imaging could allow for preemptive interventions or closer monitoring in at-risk pediatric patients (9, 14).

Notably, the actual incidence of incisional hernia in patients who undergo laparotomy in early childhood (particularly in elective cases) is generally considered low, as supported by both clinical experience and limited retrospective pediatric data (1, 2). However, this low observed rate may partly reflect underdiagnosis, as many patients remain asymptomatic and are not routinely followed with imaging. While our imaging findings suggest subclinical biomechanical changes that could predispose to later hernia development, the long-term clinical significance of these alterations remains uncertain and warrants follow-up into adolescence or adulthood.

Although the clinical hernia rates at 12 months were not significantly different between the two groups, the imaging findings (especially elevated stiffness and increased fatty infiltration) suggest a biomechanical environment that may predispose to future herniation. These observations underscore the need for extended follow-up beyond the 1-year mark to assess whether these subclinical changes translate into delayed hernia formation. Similar trends have been noted in adult cohorts, where elastographic markers have proven predictive of hernia development (9, 13).

### Postoperative wound complications

Layer-by-layer closure was associated with a higher seroma rate (12.9% vs. 3.3%, *p* = 0.0453), likely due to dead space formation between sutured layers or lymphatic disruption, a trade-off previously noted in studies comparing multilayered closures (6, 7). While seromas are typically manageable with conservative measures, their presence may delay healing and increase infection risk, particularly in immunocompromised children. Conversely, the lower seroma rate in Group A may reflect the more consolidated repair, though this benefit must be weighed against the aforementioned risks of stiffness and fibrosis.

Importantly, the distribution of emergency surgeries was balanced between groups, reducing the risk that the observed differences in tissue healing were due to the urgency or complexity of the index procedure.

In addition, both techniques demonstrated comparable rates of wound infection (8.3% vs. 8.06%) and dehiscence (3.3% vs. 4.8%), reinforcing that neither method is inherently superior in preventing acute postoperative complications. This finding aligns with prior meta-analyses concluding that suture technique (continuous vs. interrupted) and material selection play a more significant role in short-term outcomes than closure strategy alone (6, 17).

### The role of advanced imaging in prognostic assessment

A major strength of this study was its use of SWE and MSU to evaluate tissue healing beyond clinical examination. SWE's quantification of shear wave speed provided an objective measure of scar stiffness, while MSU identified subtle morphological changes (e.g., fatty infiltration, micro-defects) invisible to palpation. These modalities offer a paradigm shift in postoperative monitoring, enabling early detection of high-risk scars before herniation occurs (8, 11). For instance, the progressive increase in SWS observed in Group A over 12 months (from 2.5 m/s at 3 months to 6.4 m/s at 12 months) suggests a trajectory toward pathological stiffness, potentially heralding future hernia development.

This approach mirrors advancements in adult hernia research, where elastography has been used to predict recurrence risk (9, 13). In pediatric surgery, where incisional hernias may not manifest until adolescence, such tools could revolutionize follow-up protocols by identifying at-risk patients for targeted interventions (e.g., physical therapy or delayed elective repair).

#### Historical perspectives and evolving guidelines

The mass vs. layered closure debate dates back nearly a century, with early proponents of mass closure citing reduced dehiscence rates (18). However, as surgical techniques and materials evolved, layered closure gained favor for its anatomical precision, despite longer operative times (5, 7). Recent guidelines from hernia societies cautiously endorse layered closure but emphasize the need for pediatric-specific data (5). This study fills part of that gap, suggesting that while mass closure is efficient, layered closure may better preserve abdominal wall physiology.

#### Limitations and future directions

This trial's single-center design and 12-month follow-up period limit generalizability and preclude conclusions about very long-term outcomes (e.g., hernia rates in adolescence). Future multicenter studies with extended follow-up could clarify whether the biomechanical differences observed translate to clinically significant hernia risks. Additionally, investigating the impact of suture materials (e.g., absorbable vs. long-term absorbable) and adjuncts like prophylactic mesh in high-risk pediatric patients may further optimize closure strategies.

Although our 12-month follow-up period allowed for early detection of subclinical biomechanical alterations using SWE and MSU, we recognize that this duration may not capture all cases of delayed incisional hernia, particularly those that manifest during adolescence. However, the process of abdominal wall healing (including inflammation, fibroplasia, and collagen remodeling) typically completes within 3–6 months postoperatively (11, 12), during which the majority of tensile strength is restored. Moreover, most incisional hernias tend to present within the first postoperative year, especially in the absence of early wound complications. These factors support the clinical and biological validity of using a 12-month endpoint to evaluate tissue integrity. Nevertheless, the prognostic significance of these subclinical findings (such as increased tissue stiffness and fatty infiltration) requires validation through longer-term, multicenter studies to determine their correlation with clinical hernia incidence later in life.

## Conclusion

This study highlights a critical trade-off in pediatric laparotomy closure: while mass closure offers technical efficiency and comparable short-term safety, layer-by-layer closure may better preserve tissue elasticity and reduce long-term biomechanical deterioration.

Through the integration of shear wave elastography (SWE) and musculoskeletal ultrasound (MSU), we uncovered subclinical changes (such as increased tissue stiffness, fibrosis, and fatty infiltration) that may precede incisional hernia development.

These findings underscore the value of evaluating tissue-level healing in addition to clinical endpoints when assessing closure techniques. Particularly in children with anticipated long-term survival and potential for repeat surgeries, surgical strategies should aim not only for immediate closure integrity but also for preserving abdominal wall function over time.

MSU and SWE offer a non-invasive, reproducible, and scalable method to detect early biomechanical compromise, and their incorporation into routine pediatric postoperative surveillance could redefine early risk stratification and preventative care.

### Key recommendations

1. For time-sensitive surgeries, mass closure remains a viable option, but surgeons should consider long-term stiffness risks.
2. In elective cases, layer-by-layer closure may be preferable to optimize tissue healing.
3. Routine elastography/MSU follow-up could identify high-risk patients for early intervention.
4. Future research should explore longer-term outcomes and the role of emerging techniques (e.g., hybrid closures, bioengineered materials).

By bridging the gap between surgical tradition and modern biomechanical science, this study advances the pursuit of an evidence-based standard for pediatric laparotomy closure.

## Data Availability

The original contributions presented in the study are included in the article, further inquiries can be directed to the corresponding author.
