# Supplementary material for: Mass closure vs. layer-by-layer closure of transverse laparotomy in children: a randomized trial with ultrasound and elastography outcomes
Source: Front Pediatr. 2025 Aug 13;13:1655851. doi: 10.3389/fped.2025.1655851 (PMC12380667; doi:10.3389/fped.2025.1655851)
Supplement: Supplementary file 1 [file Datasheet1.docx]

# CONSORT 2010 Checklist of Information to Include When Reporting a Randomized Trial

| Section/Topic | Item No. | Checklist item | Reported on page(s) |
| --- | --- | --- | --- |
| Title and Abstract | 1a | Identification as a randomized trial in the title | Title page |
|  | 1b | Structured summary of trial design, methods, results, and conclusions | Abstract |
| Introduction | 2a | Scientific background and explanation of rationale | Introduction |
|  | 2b | Specific objectives or hypotheses | End of Introduction |
| Methods | 3a | Description of trial design (e.g., parallel, factorial), including allocation ratio | Methods – Study Design |
|  | 3b | Important changes to methods after trial commencement (with reasons) | Not applicable |
|  | 4a | Eligibility criteria for participants | Methods – Participants |
|  | 4b | Settings and locations where the data were collected | Methods – Study Design |
|  | 5 | Interventions for each group with sufficient details | Methods – Surgical Techniques |
|  | 6a | Clearly defined primary and secondary outcome measures | Methods – Outcome Measures |
|  | 6b | Any changes to trial outcomes after the trial commenced | Not applicable |
|  | 7a | How sample size was determined | Methods – Sample Size |
|  | 7b | Explanation of interim analyses/stopping guidelines | Not applicable |
|  | 8a | Method used to generate the random allocation sequence | Methods – Randomization |
|  | 8b | Type of randomization; details of any restriction (e.g., blocking, stratification) | Methods – Randomization |
|  | 9 | Mechanism used to implement the random allocation sequence (e.g., SNOSE), who generated and assigned | Methods – Allocation Concealment |
|  | 10 | Who was blinded after assignment to interventions (and how) | Methods – Blinding |
|  | 11a | Statistical methods used to compare groups for primary and secondary outcomes | Methods – Statistical Analysis |
|  | 11b | Methods for additional analyses (e.g., subgroup, adjusted) | Not applicable |
| Results | 13a | Flow of participants through each stage (a diagram is recommended) | Results + Figure 1 |
|  | 13b | For each group, numbers included in analysis and reasons for exclusions | Results |
|  | 14a | Dates defining the periods of recruitment and follow-up | Methods – Study Design |
|  | 14b | Why the trial ended or was stopped | Not applicable |
|  | 15 | A table showing baseline demographic and clinical characteristics | Results – Table 1 |
|  | 16 | Numbers analyzed for each outcome, by group | Results – Tables 2–4 |
|  | 17a | Outcomes and estimation for each group, with effect size and precision (e.g., CI) | Results – Tables + Paragraphs |
|  | 17b | Binary outcomes – presentation of both absolute and relative effect sizes | Results – Hernia rates |
|  | 18 | Results of other analyses performed (e.g., subgroup) | Discussion (limited) |
|  | 19 | Harms or unintended effects | Results – Seroma/Wound Complications |
| Discussion | 20 | Trial limitations, addressing sources of potential bias and imprecision | Discussion – Limitations |
|  | 21 | Generalizability (external validity) of the trial findings | Discussion – Conclusion |
|  | 22 | Interpretation consistent with results, balancing benefits and harms | Discussion |
| Other Information | 23 | Registration number and name of trial registry | Methods – Study Design |
|  | 24 | Where the full trial protocol can be accessed | Not specified |
|  | 25 | Sources of funding and role of funders | End of Manuscript |
